# Photobiomodulation Therapy on the Palliative Care of Temporomandibular Disorder and Orofacial/Cervical Skull Pain: Preliminary Results from a Randomized Controlled Clinical Trial

**DOI:** 10.3390/healthcare11182574

**Published:** 2023-09-18

**Authors:** Fernando Rodrigues Carvalho, Rafael Queiroz Barros, Alyne Simões Gonçalves, Sabrina Pinho Muragaki, Ana Clara Fagundes Pedroni, Karolyne Dias Carvalho Moschella Oliveira, Patrícia Moreira Freitas

**Affiliations:** 1 Department of Restorative Dentistry, Special Laboratory of Lasers in Dentistry, School of Dentistry, University of São Paulo, São Paulo 05508-000, Brazil; 2 Independent Researcher, São Paulo 04613-000, Brazil; 3 Department of Biomaterials and Oral Biology, School of Dentistry, University of São Paulo (USP), São Paulo 05508-000, Brazil; 4 The U.NIQ Clinic e Institute, São Paulo 01427-000, Brazil; 5 Faculdades Metropolitanas Unidas, São Paulo 04506-000, Brazil; 6 Align Technology, São Paulo 03178-200, Brazil

**Keywords:** temporomandibular disorder (TMDs), low-level laser therapies, photobiomodulation therapy, laser phototherapy, low-level light therapy

## Abstract

The main symptoms of temporomandibular disorders (TMDs) are pain from musculoskeletal and/or joint—in the head and neck region—and complaints of difficulty in mandibular movements. The photobiomodulation therapy (PBMT) has been reported as a promising treatment in the management of these symptoms. The objective of this research was to assess the effect of PBMT immediately after irradiation on TMDs symptoms under a prospective clinical trial, randomized, triple-blinded, placebo-controlled, and with two parallel arms. According to the RDC/TMD, maximum mouth opening (MMO) and pain in the orofacial/cervical muscles and temporomandibular joint (TMJ) were recorded. One hundred forty-five participants (71 placebo and 74 PBMT experimental) were analyzed after irradiation protocols (sham-PBMT or PBMT) at the orofacial/cervical skull musculature and at the TMJ. The results showed a reduction in the total pain score (*p* = 0.026), a reduction in the number of painful points (*p* = 0.013), and an increase in the MMO (*p* = 0.016) in the PBMT protocol group when compared to the placebo protocol (sham-PBMT). The PBMT was shown to be effective in reducing orofacial/cervical skull pain immediately after the irradiation. It is clinically relevant and should be taken into consideration by professionals who are dedicated to treating this pathology because, in addition to bringing comfort to patients who need dental treatment, it also consists of a low-cost and low technical complexity clinical approach.

## 1. Introduction

Temporomandibular disorders (TMDs) are defined as a group of pathologies involving the temporomandibular joint, masticatory muscles, and associated structures [1], and they affect 31% of the adult/elderly and 11% of children/adolescents [2]. Although the main symptom is pain originating from musculoskeletal and/or joints in the head and neck region [3], complaints of difficulty in mandibular movements and in the functions inherent to the stomatognathic system are also frequent [4]. The etiology can be explained by the biopsychosocial model [1] and has often been related to several other problems, such as systemic disorders, trauma, parafunctional habits, bruxism, sleep disorders, and stress, ultimately leading to a decreased quality of life [5]. The COVID-19 pandemic brought some changes in human behavior, significantly impacting the stomatognathic system and causing an increase in the prevalence and demand for treatment of TMDs [6,7]. The association of TMDs with psychological factors and bruxism made the individuals more susceptible to the onset, perpetuation and/or worsening of TMDs during the pandemic period [6,8,9]. It is suggested that TMDs are one of the symptoms of COVID-19 due to the increased prevalence among those infected [10]. In addition, an adverse effect was observed with the use of masks due to the increase in the activity of the masticatory muscles related to TMDs [11,12].

Due to the multifactorial etiological characteristics, treatment should be initiated by minimally invasive and reversible therapies [13,14,15,16]. Among these treatments, the use of interocclusal splints [17], transcutaneous electrical nerve stimulation (TENS) [18], cognitive behavioral therapy [19], pharmacological therapy [20], acupuncture [21], manual therapies [22], ultrasound [23], and photobiomodulation (PBMT) [24] stand out.

The PBMT with laser consists of the application of light with low power, which does not produce thermal effects and can promote increased cellular mitochondrial activity, leading to the synthesis and release of various metabolic substances involved in the process of pain, inflammation, and tissue repair [25,26,27,28]. In TMDs, it is expected that these effects obtained with PBMT result in increased maximum mouth opening (MMO) and reduced pain caused during the performance of stomatognathic system functions [29].

The characteristics of non-invasive, having no known side effects and absence of interactions with most drugs [26], contribute to PBMT’s favorable acceptance among patients [30]. However, professionals and researchers involved in the management of these disorders should be in charge of developing effective protocols for its management using PBMT.

This paper presents the preliminary results of a clinical trial that assesses the effect of PBMT in palliative care for temporomandibular disorders and orofacial/cranial neck pain.

## 2. Objectives

Assess the effect of PBMT immediately after irradiation on pain and MMO.

## 3. Hypotheses

The hypotheses of the present clinical trial were:

- The participants who received PBMT would report, immediately after the application, less pain of musculoskeletal and/or joint origin in the head and neck region
- The participants who received PBMT would present, immediately after the application, an increase in MMO.

## 4. Materials and Methods

### 4.1. Study Design and Ethical Considerations

This research consisted of a prospective, randomized, triple-blinded (researchers in charge of irradiating the participants, participants, and statistician), placebo-controlled, two parallel arms clinical trial. The participants were allocated to one of 2 parallel groups, PBMT or placebo (sham-PBMT). It was carried out at the Special Laboratory in Lasers in Dentistry (LELO), University of Sao Paulo (USP), Brazil, from October 2016 to March 2020.

This clinical trial was approved by the Human Research Ethics Committee, School of Dentistry of the University of São Paulo (protocol #1774930, approved on 14 October 2016) and registered on the Brazilian Registry of Clinical Trials (RBR-9b6mnj, Registered on 27 March 2018). After being informed about all the details of the research, all participants signed the Free and Informed Consent Form, as required by the Brazilian National Board of Health.

### 4.2. Sample and Randomization

The sample size was calculated based on the main outcome and assuming a Type I, significance level, error of 5%, a Type II, 80% test power, error of 20%, and 50% magnitude of effect among groups [31,32]. According to the sample calculation carried out, 200 participants should have been included, but due to the COVID-19 pandemic, the research had to be interrupted for a long period (March 2020 to September 2021), and when the researchers were able to return to continue the clinical trials at LELO, they analyzed and decided to finish the research, due the possible change in the profile of those affected by TMDs. Therefore, the sample was 153 participants. Randomization was performed in 4 blocks with 50 sealed opaque envelopes (25 envelopes for the PBMT group and 25 envelopes for the placebo group) that were mixed, numbered sequentially, and each participant after inclusion received one.

### 4.3. Participants

#### 4.3.1. Inclusion Criteria

Adults of all ages were included, both genders, regardless of race or social class, with a main complaint of pain in the TMJ region and/or orofacial/cervical skull region, with or without limitation in MMO.

#### 4.3.2. Exclusion Criteria

Participants were excluded in cases of congenital problems with the involvement of the TMJ and/or orofacial and cervical skull region; neoplastic conditions; history of recent (less than 1 month) trauma at the orofacial/cervical skull region; use of any type of TMDs treatment appliances; functional orthopedic appliances or fixed and/or removable orthodontic appliances; syndromes; cleft lip and/or palatine; psychiatric disorders; severe heart problems; tooth in severely precarious conditions, such as periodontitis and/or indication for endodontic treatment; those making use of topical or systemic photosensitizing medications or pregnant women; and dermatological diseases in the region where irradiation would be performed. Participants could not use analgesics, anti-inflammatories, or any medication that could have action on TMDs.

### 4.4. Assessment of Temporomandibular Disorders Symptoms

All participants were screened using Research Diagnostic Criteria (RDC) for TMDs [33,34]. The RDC/TMD provides a standardized way to assess and diagnose TMDs, which are a group of conditions affecting the temporomandibular joint and the muscles of mastication. The criteria help clinicians and researchers categorize TMD patients into specific diagnostic groups based on their clinical signs and symptoms. The RDC/TMD is divided into Axis I, Clinical Diagnoses: This axis focuses on classifying individuals into specific diagnostic categories based on their presenting symptoms, clinical examination findings, and specific criteria related to the disorder and Axis II: Physical and Psychosocial Functioning: this axis assesses the impact of TMD on an individual’s physical and psychosocial well-being. It includes measures related to pain intensity, jaw movement limitations, and psychosocial factors such as stress and depression. Axis 1 was applied before and after each clinical session, and Axis 2 was applied before the first session and after the last session (3rd) by the same researcher. According to the RDC/TMD [33,34], muscle (intraoral and extraoral) and joint (ATM) palpation of the head and neck was performed in 15 areas on each side, and participants reported the degree of pain for each palpated area (0 = no pain; 1 = mild pain; 2 = moderate pain; 3 = severe pain). In this work, preliminary results after the first session are presented: demographic data (gender and age), number of painful points (not considering pain intensity), total pain score (sum of pain score for each point examined), MMO measurement (the measure was performed from incisal of incisive superior to incisal of incisive inferior, with a caliper) before and after the intervention—the MMO was taken without the patient feeling pain or without the pain increasing and passively without no interference to help him open his mouth.

### 4.5. Intervention

The subjects were informed about the research, and those who agreed to participate were interviewed, examined, and submitted to treatment according to the group to which they were allocated. Participants had their skin cleaned immediately before irradiation. All biosecurity precautions were taken.

All participants were examined, and pain sites were identified and noted. In the PBMT protocol, the laser was applied at predetermined points and at specific trigger points (identified during the clinical examination and differed from predetermined locations). The application of the laser was symmetrical, that is, on both sides of the face, with the same number of points, regardless of whether it was a “painful or trigger point” or not. In the placebo protocol (sham PBMT—irradiation was performed as in the PBMT protocol; however, no laser light was emitted from the tip). The orofacial/cervical clinical examination of the skull was performed by one of the researchers, who was unaware of the group to which the participant belonged. Two laser devices were used, one for the placebo protocol (sham PBMT) and the other for the PBMT protocol. The two devices were labeled with different letters (A and B), and only the researcher responsible for randomization had access to this information. The researchers who performed the laser application did not know which equipment was the active or placebo type.

The characteristics of the low-power laser equipment and the parameters considered for irradiation are depicted in Table 1.

Predefined areas where the laser was applied were: (1) temporal muscle, 3 points (1 in the anterior muscle bundle, 1 in the middle muscle bundle, and 1 in the posterior muscle bundle); (2) masseter muscle, 6 points (3 points at the origin (zygomatic arch) and 3 points at the insertion (mandibular angle)); (3) medial pterygoid muscle, a medial point located behind the retromolar triangle; (4) sternocleidomastoid muscle, 6 points (2 at the origin of the muscle, 2 at the middle portion, and 2 at the insertion); (5) pain trigger points, 1 point for each pain point diagnosed on palpation; and (6) the TMJ, 3 points (1 point in the most posterior part of the TMJ region (the introduction of the laser light must be through the external ear, positioning the beam anteriorly), 1 in the most superior portion of the TMJ, and 1 in the anterior portion of the ATM).

The number of irradiated points depended on the extra trigger points identified during the clinical examination by muscle palpation. The total of predefined points was 19 for each side; as the application was bilateral, it was considered a total minimum of 38 irradiation points per participant.

All participants were asked about whether they experienced any discomfort during or after the PBMT application.

All details of the research “methods” were published previously [35].

### 4.6. Statistical Analysis

Data collection was performed considering “Placebo” and “PBMT” protocols and assessed before and after protocol application.

Categorical data were summarized by the absolute (n) and relative (%) frequency of the total number of cases in each protocol, and quantitative data were summarized by some summary statistics, such as mean, standard deviation, median, and minimum and maximum values according to the applied protocol.

For the comparison among protocols, the chi-square test was used for categorical parameters and the independent *t*-test for quantitative parameters. The chi-square test was also applied to verify the association between two categorical parameters.

To assess the effect of the intervention, a mixed effects analysis of variance model was used, considering the patient as a random factor and the applied protocol (“placebo” and “PBMT”), as well as the evaluation period (“before” and “after” intervention), as fixed effects. In the case of statistical significance of any of the fixed factors or even of the interaction factor, Tukey’s method of multiple comparisons was applied to determine the significant differences and an interval of confidence was calculated.

All analysis was performed using Minitab statistical software, version 18.1. Statistical significance was considered for values of *p* ≤ 0.05.

## 5. Results

This research screened 291 potential participants; 138 participants were excluded after inclusion/exclusion criteria were applied. One hundred fifty-three participants were included and randomized—132 (86.3%) female and 21 (13.7%) male. The randomization was performed using 3 blocks of 50 participants and a last block of 3 participants. Participants were divided between the two protocols—placebo (sham-PBMT) and PBMT, with 77 (50.3%) allocated to the placebo group and 76 (49.7%) to the PBMT group. Age ranged from 18 to 85 years, with a mean of 42.2 years. There was no statistically significant difference regarding the distribution of demographic parameters between protocols “placebo” and “PBMT” (chi-square test *p* = 0.789 for gender, and *t*-test *p* = 0.436 for age) (Table 2 and Table 3).

**Figure 1:**
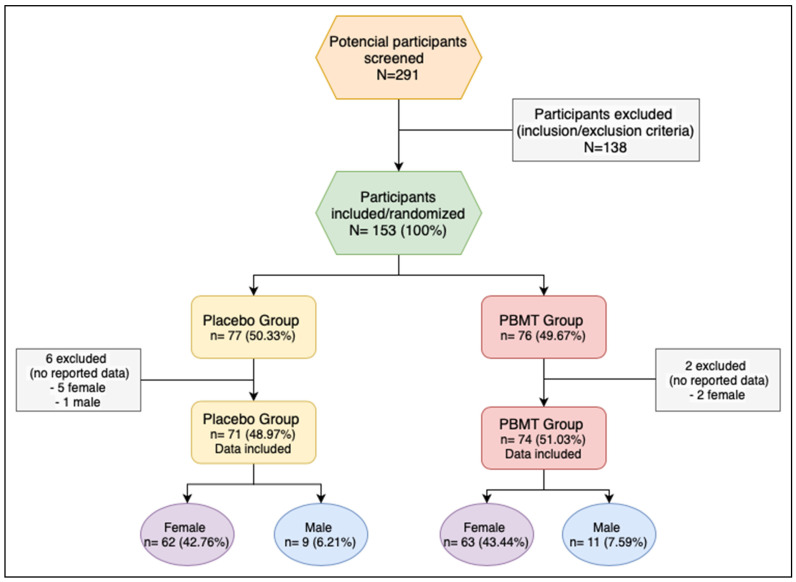
Flow chart of the study. PBMT: photobiomodulation therapy.

Eight participants were excluded after the randomization: 6 from the placebo group (5 female; 1 male) and 2 (2 female) from the PBMT group because the data was not recorded (Figure 1).

One hundred forty-five (71—placebo group; 74—PBMT group) were analyzed for the following outcomes: reduction of number of pain points, reduction of total pain score, increase in mouth opening measurement before and after the intervention.

### 5.1. Increase in MMO

The analysis of variance indicated that there was no difference between the placebo and PBMT groups regarding the MMO before the intervention (*p* = 0.768), also showing an interaction effect between the factors: protocol (“placebo” and “PBMT”) and period (“before” and “after” protocols), with *p* = 0.028 for an increase in the MMO (Table 4).

**Figure 2:**
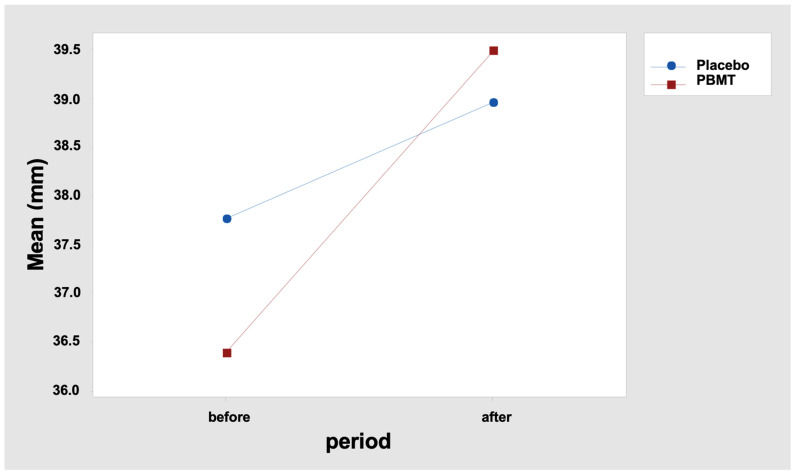
Interaction graph—MMO (mean—mm (millimeters)), period (before and after), and group (placebo or PBMT). PBMT: photobiomodulation therapy.

The results indicate that the MMO difference between the “before” and “after” periods was not similar between the two protocols. In fact, it is observed that the mean difference was 1.20 mm for the “Placebo” protocol and 3.11 mm for the “PBMT” protocol (Table 5 and Figure 2). Tukey’s multiple comparisons also reveal that the increase in the MMO after the interventions was statistically significant only for the protocol “PBMT” (Table 6 and Table 7), with *p* < 0.000 (adjusted value), confidence interval: 1.538; 4.678 (Table 7).

When an increment in the MMO ≥ 1 mm was considered as improvement, protocol “PBMT” showed a significantly higher proportion of patients with improvement (70.3%) when compared to the protocol “placebo” (50.7%), *p* = 0.016 (Table 8).

### 5.2. Reduction of Number of Tender Points

Regarding the number of tender points, the analysis of variance showed that the protocols presented similar behavior (*p* = 0.460), and both had a significant decrease in the number of points (*p* < 0.001). The groups (Placebo and PBMT) did not present differences in the number of tender points before the intervention (Table 9).

**Figure 3:**
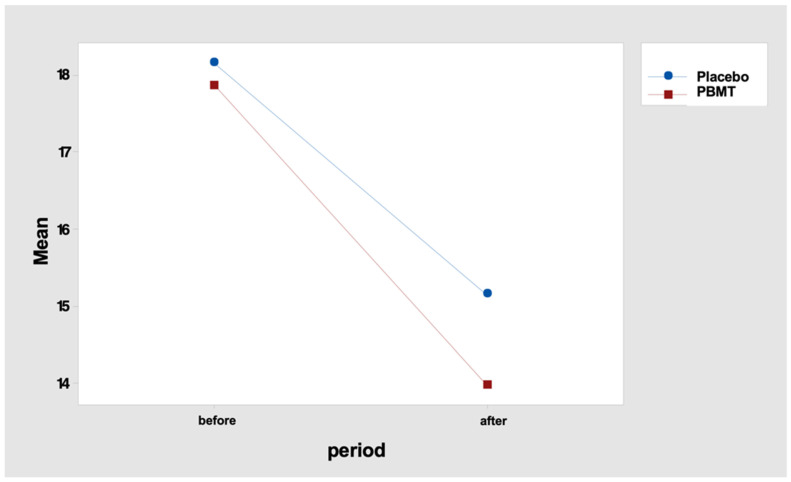
Interaction graph—number of tenders points (mean), period (before and after), and group (placebo or PBMT). PBMT: photobiomodulation therapy.

In fact, the mean difference in the number of points (“before” minus “after” period) was 3.0 for the “placebo” protocol and 3.9 for the “PBMT” protocol (Table 10 and Figure 3). Tukey’s multiple comparisons endorse this result, indicating a reduction in the number of tender points, but with no difference between the protocol, either “before” or “after” the PBMT protocol (Table 11 and Table 12).

Considering “improvement” when the patient had a reduction of at least one tender point, the PBMT protocol had a significantly higher proportion of patients with improvement (85.1%) when compared to the placebo protocol (67.6%), *p* = 0.013 (Table 13).

### 5.3. Reduction of Total Pain Score

When evaluating the total pain score, the analysis of variance indicated that there was no difference between the placebo and PBMT groups before the intervention (*p* = 0.167). It showed an interaction effect between the protocols (placebo and PBMT) and the intervention period (before and after) (*p* = 0.007) (Table 14).

**Figure 4:**
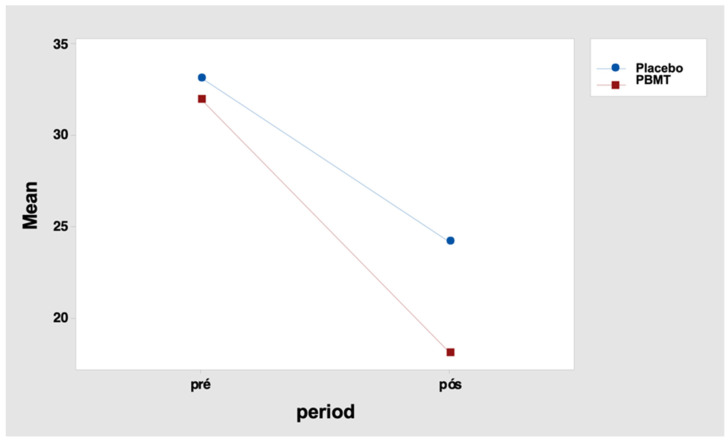
Interaction graph—total pain score (mean of data), period (before and after), and group (placebo or PBMT). PBMT: photobiomodulation therapy.

It indicates that the total pain score differences between the periods “before” and “after” were not similar between the two protocols. For the placebo protocol, this difference was 9.0 points, and for the PBMT protocol, it was 13.9 points (Table 15 and Figure 4). Tukey’s multiple comparisons also reveal that the decrease in the total score after the intervention was significant in both protocols (but greater for the PBMT protocol when compared to the placebo) (Table 16 and Table 17).

Considering “improvement” when the patient presented a reduction of at least 1 point in the pain score, it was verified that the laser protocol presented a significantly higher proportion of patients with improvement (91.9%) when compared to the placebo protocol (78.9%), *p* = 0.026 (Table 18).

There were no statistical differences between the two groups (Placebo or PBMT) in symptomatic parameters before intervention, according to Tukey’s analysis (Table 4, Table 9 and Table 14).

## 6. Discussion

This clinical trial compared the effect of the PBMT with a placebo for the treatment of TMDs. The results indicated that the PBMT was effective in reducing TMDs symptoms compared to the placebo. The main findings were the improvement of pain related to TMDs and the increase in mouth opening after laser therapy. The decrease in pain was observed with the reduction of the number of tender points and the total pain score. These findings also validate the protocol used regarding points of irradiation and dosimetric parameters, as it proved to be effective, promoting the desired results.

Although other studies [36,37] have already presented predominantly larger samples of women, this sample presents a high proportion between women and men (6.2:1). It is believed that the higher proportion of women is linked to hormonal, behavioral, and emotional factors [38].

The results of this research agree with the systematic review using a meta-analysis conducted by Hanna et al. [39], which also showed clinical improvement in participants submitted to PBMT. This comparison aimed to highlight the favorable outcomes associated with PBMT’s application in TMD treatment. However, due to the heterogeneous parameters found in the clinical studies included in the referred systematic review, it was decided not to focus on the laser dosage but on the positive result when considering the management of TMDs with PBMT. Furthermore, we understand that not only dosage set at the equipment influences the results; we should consider factors related to the patient’s (such as skin phototype), frequency of PBMT sessions, number of points irradiated, etc.

The sites for irradiation were chosen according to the main structures involved in TMDs: the cranio-orofacial and cervical muscles and the TMJ. In an attempt to create a protocol with pre-established points, irradiation was performed regardless of the degree of pain presented during the physical examination. In some cases where pain points—different from the pre-established points—were found, extra points were added for the application of the protocol. The application was carried out symmetrically, that is, on both sides, as the incorrect operation of one side would cause overload on the opposite side, which could cause late pain and negatively affect clinical outcomes.

With respect to the chosen dosimetry, which includes a light source, power, wavelength, energy per point, emission mode, and equipment tip-tissue distance, previous studies in the field of PBMT for TMDs management were considered [39,40,41,42]. The photobiomodulation therapy is dependent on dosage. Specifically, if we employ either a lower or higher energy amount per point than the ideal dose, the desired outcome will not be achieved [43]. Regrettably, the optimal dose remains unknown for certain pathologic conditions, such as TMDs [39]. In this clinical trial, apart from presenting the outcomes concerning the results of PBMT on TMDs, it also demonstrates the effectiveness of the employed dosimetric parameters.

The FBM, through photochemical, photophysical, and photobiological intra and extra-cellular processes, causes the effects of analgesia, inflammatory modulation, and induction of the tissue repair process [25,26,27,28]. The mechanism of action of PBM using a low-power laser in analgesia is not fully understood, but it is believed that the light alters the potential of the neuronal membrane. Consequently, the transmission of painful nerve impulses decreases the amount of algogenic substances, improves perfusion reducing edema/microedemas that may be compressing nerve endings, and also acts by increasing endogenous endorphins and anti-inflammatory cytokines [39,44]. The reduction of pain caused by PBMT should promote the observed effect of muscle relaxation, providing an increase in MMO [45].

When considering the limits of this clinical trial, it is important to emphasize that the initially proposed sample was not reached (N = 200). This was due to the lockdown imposed due to the COVID-19 pandemic. When the clinical trial could be resumed, we analyzed the possibility that the participants who would be included had a different TMDs etiological profile [6,8,9,10,11,12], so the study was closed with a sample of 153 participants, all included before the COVID-19 pandemic. Despite the reduction in sample size, we believe that the potential for obtaining a result due to chance was ruled out.

None of the research participants reported any discomfort or adverse effects during or after the application of PBMT. This outcome reinforces previous research findings and confirms a good acceptance of this therapy by the participants [26,30].

In this paper, the preliminary results of this clinical trial were presented; however, other data were collected regarding aspects of TMDs, such as sleep, quality of life, psychological profile, and association with malocclusion. In the future, these data will be analyzed, correlated with the adoption of PBMT, and disclosed in order to have more information about the positive impact of PBMT adoption on those affected by TMDs.

## 7. Conclusions

It is concluded that PBMT was effective in reducing general orofacial and neck pain. An improvement of symptoms associated with TMDs, specifically a reduction of total pain score, reduction of the number of painful points, and increase in mouth opening, was observed.

This result is clinically relevant and should be considered by professionals dedicated to treating this pathology because it also consists of a low-cost and low-complexity technique in addition to bringing comfort to patients who need dental treatment.

## Figures and Tables

**Table 1:** Dosimetric parameters. ^1^ cm^2^: Square centimeters.

Dosimetric Parameters	
Light source	Laser
Power (watts)	0.1
Wavelength (nanometers)	808 (Infrared)
Time per point (seconds)	30
Energy per point (joules)	3.0
Emission mode	Continuous
Equipment tip-tissue distance	Contact
Spot area (cm^2^) ^1^	0.028
Energy density (joules/cm^2^) ^1^	107.14
Power density (watts/cm^2^) ^1^	3.57

**Table 2:** Distribution between age and protocols placebo and PBMT. SD: standard deviation; Min: minimum; Max: maximum.

	Protocol	N	Mean	SD	Min.	Median	Max.
Age (years)	Placebo	77	41.30	13.37	19.00	40.66	71.30
	PBMT	75	43.13	15.38	18.73	41.67	85.84

**Table 3:** Distribution between gender and protocols placebo and PBMT. PBMT: photobiomodulation therapy.

		Protocol		
		Placebo	PBMT	Total
Female	n	67	65	132
	%	87.01	85.53	86.27
Male	n	10	11	21
	%	12.99	14.47	13.73
Total	n	77	76	153
	%	100.00	100.00	100.00

**Table 4:** MMO—Fixed Effects Tests. DF-Num: degree of freedom in the numerator; DF-Den: degree of freedom in the denominator. PBMT: photobiomodulation.

Term	DF-Num	DF-Den	F Value	*p* Value
Period (before/after)	1.00	143.00	24.89	0.000
Protocol (PBMT/placebo)	1.00	143.00	0.09	0.768
Protocol × period	1.00	143.00	4.90	0.028

**Table 5:** MMO: maximal mouth opening before and after intervention; SD: standard deviation; Min: minimum; Max: maximum. PBMT: photobiomodulation.

	Protocol	N	Mean	SD	Min.	Media	Max.
MMO before intervention	Placebo	71	37.76	8.44	13.00	40.00	54.00
	PBMT	74	36.38	9.52	16.00	35.50	60.00
MMO after intervention	Placebo	71	38.95	8.24	19.00	39.00	59.00
	PBMT	74	39.49	9.96	16.00	39.00	65.00
MMO (after—before)	Placebo	71	1.197	4.63	−13.00	1.00	14.00
	PBMT	74	3.10	5.68	−9.00	2.00	17.00

**Table 6:** MMO—Tukey multiple comparisons (95% confidence). Means that do not share a letter are significantly different. PBMT: photobiomodulation.

Protocol × Period	N	Mean	Grouping	
PBMT × after	74	39.48	A	
Placebo × after	71	38.95	A	B
Placebo × before	71	37.76	A	B
PBMT × before	74	36.37		B

**Table 7:** MMO: Simultaneous Tukey tests for differences in means. Individual confidence level = 98.97%. DM: difference of mean; SE: standard error; DF: degree of freedom; CI: confidence intervals. PBMT: photobiomodulation.

Difference of Protocol × Period Levels	DM	SE of Difference	DF	CI (95% Simultaneous)	T-Value	*p*-Value Adjusted
(Placebo after)—(Placebo before)	1.19	0.61	143.00	(−0.40; 2.80)	1.940	0.215
(PBMT before)—(Placebo before)	−1.38	1.51	168.25	(−5.31; 2,54)	−0.920	0.796
(PBMT after)—(Placebo before)	1.73	1.51	168.25	(−2.20; 5.65)	1.140	0.663
(PBMT before)—(Placebo after)	−2.58	1.51	168.25	(−6.50; 1.34)	−1.710	0.323
(PBMT after)—(Placebo after)	0.53	1.51	168.25	(−3.39; 4.45)	0.350	0.985
(PBMT after)—(PBMT before)	3.10	0.60	143.00	(1.53; 4.67)	5.150	0.000

**Table 8:** MMO; chi-square test: *p* = 0.016. PBMT: photobiomodulation therapy.

		Placebo	PBMT	Total
Improved	n	36	52	88
	%	50.70	70.27	60.69
No-improve	n	35	22	57
	%	49.30	29.73	39.31
Total	n	71	74	145
	%	100	100	100

**Table 9:** Number of tender points—fixed effects tests. DF-Num: degree of freedom in the numerator; DF-Den: degree of freedom in the denominator. PBMT: photobiomodulation therapy.

Term	DF-Num	DF-Den	F Value	*p* Value
Protocol (PBMT/placebo)	1.00	143.00	0.28	0.597
Period (before/after)	1.00	143.00	33.09	0.000
protocol × period	1.00	143.00	0.55	0.460

**Table 10:** Number of tender points: before and after the intervention; SD: standard deviation; Min: minimum; Max: maximum. PBMT: photobiomodulation therapy.

	Protocol	N	Mean	SD	Min	Median	Max
Tender points before	Placebo	71	18.16	7.94	2	19	30
	PBMT	74	17.87	7.46	4	18	30
Tender points after	Placebo	71	15.15	8.30	0	15	30
	PBMT	74	13.97	11.95	0	12	84
Tender points (before—after)	Placebo	71	3.01	4.61	−7	2	18
	PBMT	74	3.91	9.07	−56	5	22

**Table 11:** Number of tender points—Tukey multiple comparisons (95% confidence). Means that do not share a letter are significantly different.

Protocol × Períod	N	Mean	Grouping		
Placebo × before	71	18.16	A		
PBMT × before	74	17.87	A	B	
Placebo × after	71	15.15		B	C
PBMT × after	74	13.97			C

**Table 12:** Number of tender points: Simultaneous Tukey tests for differences in means. Individual confidence level = 98.97%. DM: difference of mean; SE: standard error; DF: degree of freedom; CI: confidence intervals; PBMT: photobiomodulation therapy.

Difference of Protocol × Period Levels	DM	SE of Difference	DF	CI (95% Simultaneous)	T-Value	*p*-Value Adjusted
(Placebo after)—(Placebo before)	−3.01	0.85	143.00	(−5.24; −0.78)	−3.51	0.003
(PBMT before)—(Placebo before)	−0.29	1.51	194.81	(−4.22; 3.64)	−0.19	0.997
(PBMT after)—(Placebo before)	−4.20	1.51	194.81	(−8.13; −0.26)	−2.77	0.032
(PBMT before)—(Placebo after)	2.72	1.51	194.81	(−1.21; 6.66)	1.80	0.278
(PBMT after)—(Placebo after)	−1.18	1.51	194.81	(−5.12; 2.75)	−0.78	0.863
(PBMT after)—(PBMT before)	−3.90	0.84	143.00	(−6.09; −1.71)	−4.64	0.000

**Table 13:** Number of tender points. Chi-square test: *p* = 0.013. PBMT: photobiomodulation therapy.

		Placebo	PBMT	Total
Improved	n	48	63	111
	%	67.61	85.14	76.55
No-improved	n	23	11	34
	%	32.39	14.86	23.45
Total	n	71	74	145
	%	100	100	100

**Table 14:** Total pain score—fixed effects tests. DF-Num: degree of freedom in the numerator; DF-Den: degree of freedom in the denominator; PBMT: photobiomodulation therapy.

Termo	DF-Num	DF-Den	F Value	*p*-Value
Protocol (placebo/PBMT)	1.00	143.00	1.93	0.167
Period (before/after)	1.00	143.00	163.40	0.000
protocol × períod	1.00	143.00	7.57	0.007

**Table 15:** Total pain score: before and after the intervention; SD: standard deviation; Min: minimum; Max: maximum.

	Protocol	N	Mean	SD	Min	Median	Max
Score before	Placebo	71	33.13	18.64	2.00	30.00	79.00
	PBMT	74	31.96	17.20	4.00	29.50	83.00
Score after	Placebo	71	24.15	17.20	0.00	22.00	73.00
	PBMT	74	18.07	12.93	0.00	16.00	58.00
before—after	Placebo	71	8.97	10.67	−7.00	6.00	36.00
	PBMT	74	13.89	10.86	−16.00	12.00	44.00

**Table 16:** Total pain score -Tukey multiple comparisons (95% confidence). Means that do not share a letter are significantly different. PBMT: photobiomodulation.

Protocol × Períod	N	Mean	Grouping	
Placebo before	71	33.12	A	
PBMT before	74	31.95	A	
Placebo after	71	24.15		B
PBMT after	74	18.06		B

**Table 17:** Total pain score: Simultaneous Tukey tests for differences in means. Individual confidence level = 98.97%. DM: difference of mean; SE: standard error; DF: degree of freedom; CI: confidence intervals; PBMT: photobiomodulation therapy.

Difference of Protocol × Period Levels	DM	SE of Difference	DF	CI (95% Simultaneous)	T-Value	*p*-Value Adjusted
(Placebo after)—(Placebo before)	−8.97	1.28	143.00	(−12.29; −5.65)	−7.02	0.000
(PBMT before)—(Placebo before)	−1.17	2.76	176.14	(−8.34; 6.00)	−0.42	0.974
(PBMT after)—(Placebo before)	−15.06	2.76	176.14	(−22.23; −7.89)	−5.46	0.000
(PBMT before)—(Placebo after)	7.80	2.76	176.14	(0.63; 14.97)	2.83	0.027
(PBMT after)—(Placebo after)	−6.09	2.76	176.14	(−13.26; 1.08)	−2.21	0.126
(PBMT after)—(PBMT before)	−13.89	1.25	143.00	(−17.15; −10.64)	−11.10	0.000

**Table 18:** Total pain score. Chi-square test: *p* = 0.026. PBMT: photobiomodulation therapy.

		Placebo	PBMT	Total
Improved	n	56	68	124
	%	78.87	91.89	85.52
No-improved	n	15	6	21
	%	21.13	8.11	14.48
Total	n	71	74	145
	%	100.00	100.00	100.00

## Data Availability

The available data is contained within the article. We will use this and other data for future publications.
